# Editorial: The neuroscience of advancing age

**DOI:** 10.3389/fnagi.2023.1195785

**Published:** 2023-04-14

**Authors:** Ann-Maree Vallence, Rachael D. Seidler, Mitchell Ryan Goldsworthy, John G. Semmler, George M. Opie

**Affiliations:** ^1^School of Psychology, College of Health and Education, Murdoch University, Murdoch, WA, Australia; ^2^Centre for Healthy Ageing, Health Futures Institute, Murdoch University, Murdoch, WA, Australia; ^3^Department of Applied Physiology and Kinesiology, University of Florida, Gainesville, FL, United States; ^4^Discipline of Physiology, School of Biomedicine, The University of Adelaide, Adelaide, SA, Australia; ^5^Hopwood Centre for Neurobiology, Lifelong Health Theme, South Australian Health and Medical Research Institute (SAHMRI), Adelaide, SA, Australia

**Keywords:** NIBS, healthy aging, pathological aging, neuroimaging, neuromodulation

While some older adults retain physical and mental capabilities comparable to individuals that are decades younger, others may be incapable of self-care. Understanding the factors that determine where an individual will find themselves on this spectrum, particularly those that may be modifiable, is therefore critical. In pursuit of this goal, the current Research Topic focussed specifically on how changes within the brain contribute to both healthy and pathological aging.

## Changes in brain structure and network function

Changes to brain structure are a hallmark of aging that strongly influences how we age; reductions in white matter (WM) integrity are one example that has been suggested to underpin many age-related functional deficits (for example, [Madden et al., 2004; Van Petten et al., 2004; Kerchner et al., 2012]). To further investigate this, Yan et al. assessed how age-related changes in WM contribute to the ability of older adults to perceive global motion. This study used diffusion tensor imaging (DTI) to identify several specific white matter tracts where age-related reductions in integrity predicted reduced global motion perception. This was suggested to support the “disconnection hypothesis” of cognitive aging. Using a slightly different approach, Du et al. instead leveraged magnetic resonance histology (Johnson et al., 2022) to study genetic and age effects on brain volume within auditory areas. The volume of twelve auditory regions was quantified in 104 young and older animals from the BXD family of recombinant inbred mice. Interestingly, genotype was found to influence volume of auditory areas, and this effect differed in older animals. This important work provides a platform for better understanding age effects on auditory function, degradation of which is associated with many negative outcomes.

While local changes in specific brain structures are clearly important, considering how alterations to multiple structural elements interact to drive brain aging is also critical. To incorporate this, Hupfeld et al. recorded several indices of brain structure (including volumetric, surface and WM integrity) to report a number of deficits having a complex relationship with reduced performance in a dual-task walking paradigm. In contrast, Vaughan et al. instead utilized machine-learning to quantify how an individual's chronological age differed from their brain-predicted age (as an indicator of accelerated or decelerated brain aging). They then demonstrated that accelerated brain aging moderated the relationship between leg strength and mobility.

One important factor often overlooked in studies of brain aging is the large differences in hormones between females and males across the lifespan. To assess the potential influence this may have on brain imaging studies, Hicks et al. examined associations between sex steroid hormones and age-network relationships in both males and females, focusing on network segregation. Although network segregation was not associated with hormone levels in females, network segregation in the cerebellar-basal ganglia and salience networks showed a complex relationship with age. Given their role in cognition and balance, it is important to understand how age-related changes in segregation within these networks drive behavior.

## Modulating and testing function in older adults

Modulation of brain activity represents a promising avenue for improving how we age, and one approach to achieving this is to use non-invasive brain stimulation (NIBS). For example, Greeley et al. investigated how anodal transcranial direct current stimulation (atDCS) over prefrontal cortex influenced sequence learning in older adults. While increased performance was expected, atDCS instead reduced learning, possibly due to the timing of the intervention. These findings add to a growing body of literature demonstrating the need for optimized NIBS interventions in older adults.

As an alternative example of approaches to brain modulation, acupuncture has shown an ability to modify aberrant activity in patients with mild cognitive impairment (MCI), although the outcomes have been variable. Consequently, the meta-analysis by Ma and colleagues compiled the evidence examining the influence of acupuncture on functional MRI measures in MCI patients. Across studies, acupuncture was found to increase activity within several brain areas, with changes in thalamic areas associated with cognitive function. While supporting the potential utility of acupuncture in MCI, the authors also recognize confirmation is needed from more rigorous RCT studies. However, the outcomes of this study nonetheless demonstrate the potential utility of alternative approaches for influencing the aging brain. The study by Ma et al. further demonstrates this within the context of AD. This study investigated the therapeutic mechanisms of Cordycepin, a nucleoside adenosine analog derived from traditional Chinese medication that has shown anti-AD properties. Using network pharmacology and molecular docking methods, five genes (AKT1, MAPK8, BCL2L1, FOXO3, and CTNNB1) potentially serving as targets of Cordyceptin were identified. These in-silico findings provide novel insights for developing optimal treatments for AD.

While modulating function represents a core aim of many studies in aging neuroscience, assessing function remains a fundamental necessity. However, existing approaches are often time consuming or require specialist clinicians. To address this, Cattaneo et al. validated the “Guttman Cognitest” – a self-administered digital cognitive assessment requiring ~20 minutes – against conventional paper-and-pencil tests in a middle-aged cohort. Principal component analysis revealed three factors consistent between test types (memory, executive function, and visuomotor / visuospatial function), although there was some variance in how specific subtests loaded onto each factor. Furthermore, performance showed the expected negative association with age and positive association with years of education. The authors concluded that this digital assessment is appropriate for measuring cognitive function in large samples of middle-aged adults.

## The role of systemic factors in brain aging

Consideration of the neuroanatomical and neurophysiological effects of age is often restricted to the context of the central nervous system (CNS). In contrast, interactions with the systemic environment may also contribute to age-related changes within the brain. For example, inflammation is increased in older adults, which may contribute to age-related cognitive deficits (Sartori et al., 2012). While this inflammatory state (and associated cognitive deficits) is often considered to develop with age, Ni and colleagues instead investigated if early-life events are also important. Specifically, a mouse model was used to show that maternal immune activation increases inflammation and exacerbates age-related cognitive deficits in offspring. Importantly, the same study also demonstrated that the influence of the prenatal environment could be mitigated by life-long environmental enrichment, demonstrating the importance of lifestyle on how we age. While interesting, the role played by inflammation during aging, particularly with respect to pathological aging, remains unclear. To clarify this, Leonardo and Fregni performed a systematic review and meta-analysis examining the relation between inflammatory cytokines and cognitive impairment from 79 studies in people with MCI or AD. Their findings suggested higher levels of several inflammatory cytokines in MCI and AD, in addition to greater risk of cognitive decline with high interleukin-6 (IL-6) levels. These outcomes indicate that increased cytokine levels within the CNS may be a potential therapeutic target for AD.

Hypertension represents another systemic condition prevalent in older adults. While its relationship with cognitive function in age has been demonstrated, Chen et al. instead assessed how hypertension may contribute to increased rates of depression and sleep disturbance in the elderly. Relative to normotensive older adults, those with hypertension showed poorer cognitive function and sleep quality, in addition to higher depression. Furthermore, mediation analysis suggested sleep quality and depression partially mediate the influence of hypertension on cognitive function. Consequently, sleep quality and depression represent potential targets in a multifaceted approach to improving cognitive function in older adults.

## Conclusion

In conclusion, this Research Topic reports novel findings from 12 articles (involving >80 authors) covering a broad range of topics within the neuroscience of advancing age. This includes important new information about brain imaging, brain modulation, functional assessment, and the interaction between systemic and central factors. On the one hand, the breadth of this collection demonstrates the enormity of the challenge faced by the field. However, on the other hand, it's constellation of insightful approaches demonstrates that this challenge will be faced with enthusiasm.

## Author contributions

GO drafted the manuscript. All authors critically revised and approved the manuscript.
